# Gut microbiota–metabolite interactions in cisplatin-induced acute kidney injury in rats

**DOI:** 10.1186/s12866-026-04927-7

**Published:** 2026-03-11

**Authors:** Jiawei Wang, Yongjun Cui, Yaming Li, Junhua Yuan, Dongchuan Li, Kunjing Gong, Shaohua Li, Yaqin Wang

**Affiliations:** 1 School of Basic Medicine, Qingdao University, Qingdao, 266071 Shandong Province China; 2 School of Life Sciences and Health, University of Health and Rehabilitation Sciences, Qingdao, 266113 Shandong Province China; 3 Laboratory of Nephrology & Department of Nephrology, Qingdao Municipal Hospital, Qingdao, 266011 Shandong Province China; 4 Department of Nephrology, The Affiliated Hospital of Qingdao University, Qingdao, 266003 Shandong Province China; 5 Department of Special Medicine, School of Basic Medicine, Qingdao University, Qingdao, 266071 Shandong Province China; 6 Shandong Second Medical University Affiliated Qingdao Eighth People’s Hospital, Qingdao, 266041 Shandong Province China; 7 Department of Nephrology, Qilu Hospital, Cheeloo College of Medicine, Shandong University, Qingdao, 266011 Shandong Province China; 8 Department of Cardiology, The Affiliated Hospital of Qingdao University, Qingdao, 266003 Shandong Province China

**Keywords:** Acute kidney injury (AKI), Cisplatin, Gut microbiota, Metabolomics, Gut–kidney axis

## Abstract

**Background:**

The interplay between the gut microbiota and metabolites in the early stages of cisplatin-induced acute kidney injury (AKI) remains largely unexplored, especially in the early stages. This study aimed to identify the gut microbiota and metabolomic characteristics following cisplatin-induced AKI and to investigate the underlying mechanisms involved.

**Results:**

Male Wistar rats were randomly assigned to a control group (NC) or a cisplatin-induced AKI group (Cis). The gut microbiota composition was analysed using 16 S rRNA sequencing, and faecal metabolomic profiles were characterized using untargeted metabolomics (UPLC‒MS/MS). The relationships among serum creatinine (SCr), blood urea nitrogen (BUN), faecal metabolites, and the gut microbiota were investigated to identify potential biomarkers and therapeutic targets for AKI. The functional impact of the identified metabolites was further assessed in HK-2 human renal tubular epithelial cells using Cell Counting Kit-8 (CCK-8) and flow cytometry assays. Cisplatin administration induced significant dysbiosis of the gut microbiota, altering its composition. The Cis group was enriched in proinflammatory genera such as *Enterococcus* and *Anaerostipes*, whereas the NC group was enriched in potentially beneficial genera such as *Brotonthovivens*, whose abundance was negatively correlated with the SCr and BUN levels. Concurrently, 20 differential faecal metabolites, including elevated dehydroepiandrosterone (DHEA) and N-acetylaspartic acid (NAA), were significantly correlated with impaired renal function. Correlation network analysis further revealed intricate associations between specific bacterial abundances and metabolite levels: *Enterococcus* was positively correlated with DHEA and NAA but negatively correlated with adenosine, guanine, and linoleic acid, whereas *Brotonthovivens* exhibited the opposite pattern. Moreover, analysis of colonic tissue revealed significant downregulation of the expression levels of the tight junction proteins ZO-1 and occludin, indicating impaired intestinal barrier integrity. Based on these integrated in vivo findings, targeted in vitro validation in HK-2 cells demonstrated that adenosine significantly attenuated cisplatin-induced cytotoxicity and apoptosis, whereas NAA exacerbated these injuries.

**Conclusions:**

Our findings demonstrated that cisplatin-induced AKI significantly reshaped the gut microbiota and faecal metabolome in rats. This study demonstrates potential interactions between specific gut microbes and host metabolites during AKI progression, offering novel insights into the gut–kidney axis and highlighting potential microbial and metabolic targets for future therapeutic interventions.

**Supplementary Information:**

The online version contains supplementary material available at 10.1186/s12866-026-04927-7.

## Introduction

Cisplatin is a widely used platinum-based chemotherapeutic agent that exerts its antitumour effect by forming DNA crosslinks, leading to apoptosis in rapidly dividing cells [1]. Despite its clinical efficacy, cisplatin therapy is often compromised by nephrotoxicity, with up to 30% of patients receiving high-dose regimens developing acute kidney injury (AKI), a condition associated with increased morbidity and poor prognosis [2–4]. The pathogenesis of cisplatin-induced AKI is multifactorial and involves oxidative stress, mitochondrial dysfunction, apoptosis, vascular injury, and inflammation [5–7]. These pathological changes lead to the abrupt accumulation of nitrogenous waste products, reduced urine output, and, in severe cases, high mortality rates [8]. Although oxidative stress and inflammation have been established as key contributors, emerging evidence highlights the pivotal role of the intestinal microbiota in modulating renal injury.

In recent years, the gut–kidney axis, a bidirectional communication system in which the gut microbiota and its metabolites influence renal function and vice versa, has attracted increasing attention [9]. Dysbiosis of the gut microbiota has been implicated in AKI pathogenesis, not only through shifts in the abundance of critical bacterial taxa but also via alterations in the landscape of microbial metabolites. For instance, a decrease in short-chain fatty acids (SCFAs) can impair intestinal barrier integrity, increase gut permeability, and facilitate bacterial translocation [10, 11]. These changes activate systemic inflammatory responses through pathways such as TLR4/NF-κB signalling, ultimately exacerbating renal injury [12, 13]. Preclinical studies have demonstrated that cisplatin administration disrupts the gut microbiota, depleting beneficial bacteria such as *Lactobacillus* and *Bifidobacterium* while enriching for opportunistic pathogens [12]. These perturbations are often accompanied by decreased production of protective metabolites and an impaired intestinal barrier, potentially fuelling the inflammatory cascade in AKI [14]. Despite these advances, the precise interplay between cisplatin-induced microbial dysbiosis, attendant metabolite alterations, and the progression of renal injury remains inadequately defined. Currently, strategies such as probiotics, prebiotics, and dietary modifications are being explored as potential therapeutic avenues to alleviate the effects of cisplatin on the gut–kidney axis [15, 16]. Nevertheless, further research is needed to elucidate the precise role of the gut microbiota and its metabolites in renal injury resulting from cisplatin-induced AKI.

Therefore, this study employed an integrated approach involving 16 S rRNA gene sequencing and untargeted metabolomics (UPLC‒MS/MS) to comprehensively characterize alterations in the gut microbiota and faecal metabolites in a rat model of cisplatin-induced AKI. We aimed to (1) identify differentially abundant bacterial taxa and metabolites and (2) elucidate potential interactions within the gut microbiota–metabolite network. Our results provide new perspectives on the mechanisms of the gut–kidney axis in cisplatin-induced AKI and may reveal potential microbiota-targeted therapeutic strategies to mitigate nephrotoxicity.

## Materials and methods

Cisplatin (PHR1624) was purchased from Merck Millipore.

### Experimental animals

**Fig. 1:**
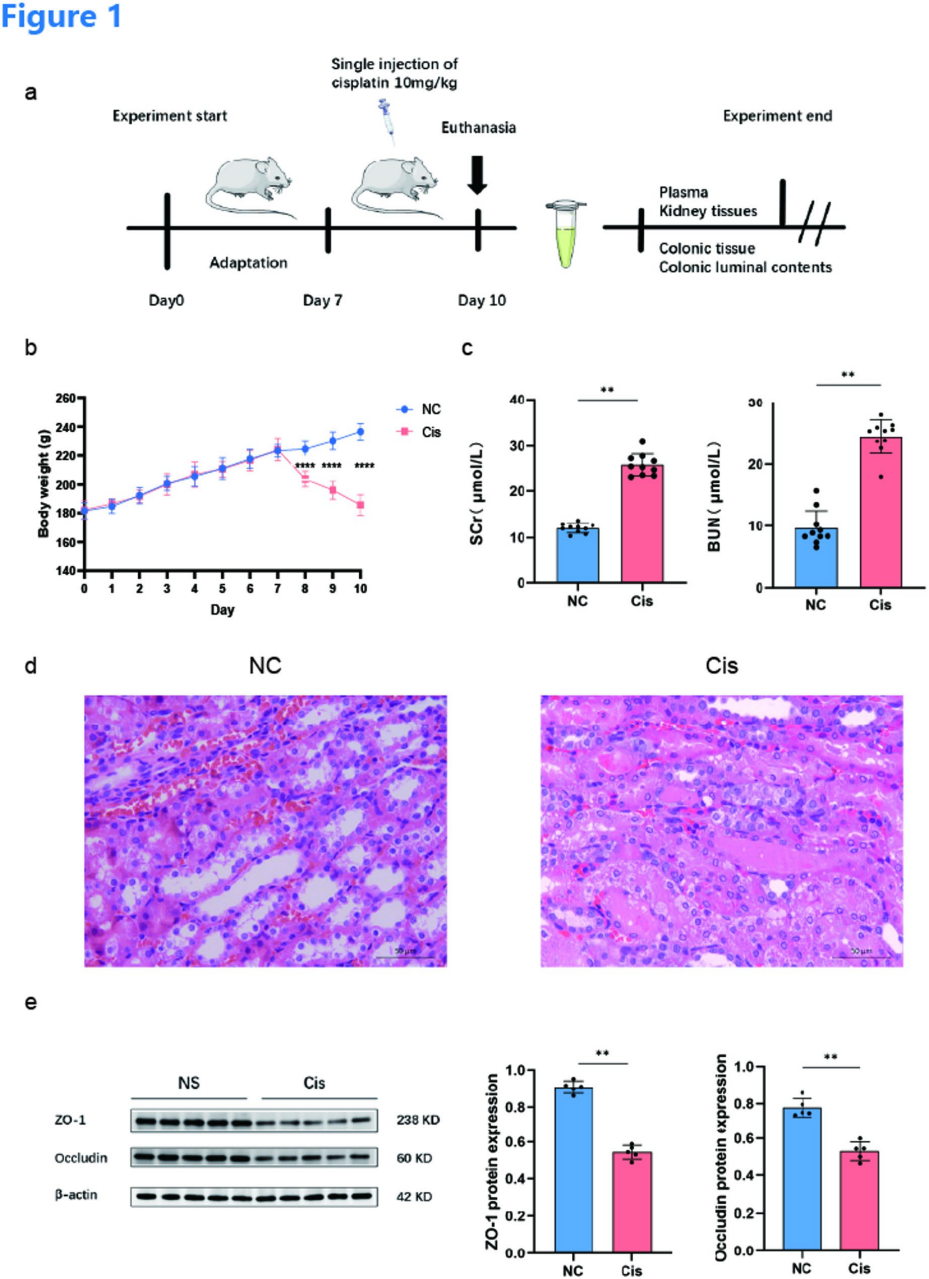
Development and validation of the cisplatin-induced acute kidney injury (AKI) rat model. **a** Experimental protocol timeline: a 7-day acclimatization period followed by a single intraperitoneal injection of cisplatin (10 mg/kg) on day 7, with tissue collection conducted on day 10. **b** Body weight variations over the 10-day period. The Cisplatin-treated group (Cis) demonstrated significant weight loss following cisplatin administration, whereas the normal control group (NC) exhibited continuous weight gain. **c** Serum creatinine (SCr) and Blood urea nitrogen (BUN) levels in the two groups. **d** Representative H&E-stained kidney tissue sections from rats in the NC and Cis groups. **e** ZO-1 and occludin expressions were detected by western blot in ileum tissue. SCr, serum creatinine; BUN, blood urea nitrogen

Male Wistar rats weighting between 180 and 200 g were obtained from Beijing Vital River Laboratory Animal Technology Co., Ltd. (Beijing, China). Protocols involving animals received approval from the Ethics Committee at Fuwai Cardiovascular Hospital (Permit Number: 2011 − 341). All the animals were fed under SPF conditions. The rats (*n* = 20) were randomized into two groups: a control group (NC, *n* = 10) and a cisplatin (10 mg/kg dissolved in saline) group (Cis, *n* = 10). Following a 7-day acclimatization period, cisplatin was injected intraperitoneally once into the rats on Day 7. Ten rats were sacrificed at 72 h after cisplatin injection, and ten rats in the control group were sacrificed together with the other rats on Day 10. The rats were euthanized via intraperitoneal injection of 2% sodium pentobarbital (150 mg/kg). Following the injection, death was verified by the lack of corneal and pinch reflexes, the cessation of spontaneous breathing, and total muscle relaxation. The plasma, colonic luminal contents, kidney tissues and colonic tissue of the rats were gathered and processed for untargeted metabolomic, 16 S rRNA sequencing, and histological analyses (Fig. 1a).

### 16 S rRNA gene sequencing and bioinformatics analysis

Rat stool DNA was extracted using a MagPure Stool DNA KF kit B (Magen, China) according to the manufacturer’s guidelines. The DNA was quantified with a Qubit Fluorometer using a Qubit dsDNA BR Assay kit (Invitrogen, USA) and then kept at − 80°C for subsequent analysis. PCR amplification of the 16S rRNA gene was performed using primers 341F (5’-ACTCCTACGGGAGGCAGCAG-3’) and 806R (5’-GGACTACHVGGGTWTCTAAT-3’), which target the hypervariable V3–V4 region. The amplification products were subsequently purified to construct the sequencing library. The validated libraries were sequenced with an Illumina MiSeq platform (BGI, Shenzhen, China) to generate 2 × 300 bp paired-end reads.

Raw 16 S rRNA gene sequencing reads were spliced and quality checked with FLASH to eliminate low-quality reads and adapters [17]. OTU clustering was performed with UPARSE at 97% sequence similarity, and chimaeras were removed using UCHIME. Taxonomic annotation was carried out using the RDP Classifier against the Greengenes database. Downstream analyses included alpha/beta diversity assessment, PCoA (weighted UniFrac distance), LEfSe for differential taxa screening, and microbial functional prediction via PICRUSt2.

### Analysis of untargeted metabolomics

Faecal metabolite analysis was performed using untargeted metabolomics UPLC‒MS/MS. Ten frozen stool samples (25 mg) were packed separately with steel beads. To extract metabolites from the samples, 800 µL of a methanol–acetonitrile–H_2_O mixture (2:2:1, v: v:v), was added, then vortexed and centrifuged. Next, 600 µl of the supernatant was collected, and the leftover pellets were extracted again using 600 µl of methanol–H_2_O (1:9, v: v). After obtaining the faecal extracts, they were obtained and centrifuged at 25,000 rpm for 15 min at 4 °C, the resulting supernatants were then carefully transferred into sample vials for LC‒MS analysis [18].

An ultrahigh-performance liquid chromatography (UPLC) system equipped with a UPLC BEH C18 column (2.1 mm * 100 mm, 1.7 μm; Waters, USA) linked to a Q Exactive Orbitrap (Thermo Fisher Scientific, USA) was utilized. For positive (ES+) and negative (ES−) modes, solvent A consisted of 0.1% formic acid and 10 mM ammonium formate, respectively. Solvent B was composed of 100% methanol with 0.1% formic acid for ES + and 95% methanol with 10 mM ammonium formate for ES−. For analysis, approximately 5 µL of each supernatant was injected at 45 °C for analysis. The gradient elution of solvent B was conducted as follows: 2%, 0–1 min; 98%, 1–9 min; 98%, 9–12 min; 98–2%, 12–12.1 min; and 2%, 12.1–15 min. The full-scan survey MS and MS/MS spectra were captured by a Thermo Q Exactive Orbitrap mass spectrometer, with ES + and ES− spray voltages being 3.8 kV and 3.2 kV, respectively. The capillary temperature was heated to 320 °C. Masses ranging from about 70 to 1050 m/z masses were obtained. The resolution for full MS and MS/MS was configured at 70,000 and 17,500, respectively.

The MS data obtained were transformed into *mzXML* using Compound Discoverer 3.1 (Thermo Fisher Scientific, USA) and analysed in the R package metaX v3.3. The data processed consists of the mass-to-charge ratio (m/z), peak intensity, and retention time (RT). To normalize the data and obtain the relative peak area, the probabilistic quotient normalization (PQN) method was applied. Any compounds with a relative peak area CV exceeding 30% in all QC samples were removed. The metabolites were identified according to their m/z values according to the BGI Library and the online HMDB, mzCloud, Chemspider and KEGG databases. To validate and confirm the metabolites, commercial reference standards were employed by comparing their retention times and MS/MS spectra.

We used principal component analysis (PCA) to assess whether the cisplatin and control groups could be separated. Partial least squares discriminant analysis (PLS-DA) was carried out to evaluate the difference in metabolic profiles between the two groups. Findings with a variable importance in projection (VIP) > 1.5, *p <* 0.05 and a fold change ≥ 1.25 or ≤ 0.83 were deemed significant for distinguishing between groups. Finally, significantly modulated metabolites were annotated through metabolic pathways in the KEGG database.

### Cell culture and treatment

Cultivation of human renal tubular epithelial cells (HK-2) (ATCC, USA) were performed in DMEM/F12 medium (Gibco, USA.), containing with 10% foetal bovine serum (Gibco, USA). The cells were grown in medium and incubated at 37 °C with 5% CO₂ to ensure ideal growth conditions. During this process, cell passage number of the cells was monitored, and the cells used in the experiment were not passaged more than three times. HK-2 cells were seeded at 3 × 10⁵ cells per well in 6-well cell culture plates.

HK-2 cells were randomly allocated into 5 groups (*n* = 6): control, cisplatin, cisplatin + NAA (1 and 2 mM), cisplatin + adenosine (50 and 100 µM), and cisplatin + linoleic acid (10 and 20 µM). The control group was cultured in complete medium for 24 h; the cisplatin group received a 20 µM cisplatin for 24 h for induction; the cisplatin + NAA group was preincubated with 1 mM or 2 mM NAA for 1 h, followed by coculture with 20 µM cisplatin for 24 h; the cisplatin + adenosine group was preincubated with 50 or 100 µM adenosine for 1 h, followed by coculture with 20 µM cisplatin for 24 h; and the cisplatin + linoleic acid group was preincubated with 10 or 20 µM linoleic acid for 1 h, followed by coculture with 20 µM cisplatin for 24 h.

### Cell viability assay

HK-2 cells were plated in 96-well plates at a concentration of 4⋅10⁴per well, and the plates were subsequently incubated at 37 °C with 5% CO₂. After the supernatant was discarded, 100 µL of fresh medium supplemented with 10 µL of CCK-8 reagent (CCK-8, Dojindo, Japan) was added, and the medium was gently shaken a few times [19]. The absorbance for each well was determined at 450 nm using a microplate reader (Molecular Devices, Germany).

### Flow cytometry

To identify apoptosis in HK-2 cells, flow cytometry was employed. The supernatant was aspirated, and the cells were gently washed with precooled phosphate-buffered saline (PBS). Trypsin was added for digestion, followed by centrifugation at 250⋅g for 5 min to collect the cell precipitate. Precooled PBS was added, and the samples were then centrifuged twice [20]. After incubating the cell suspension at room temperature in the dark for 10 min, Annexin V-APC (5 µL) and PI (5 µL) were allowed to bind to the appropriate cellular components, and apoptosis was subsequently detected by flow cytometry (Beckman, Germany).

### Western blotting

The protein expression levels of ZO-1 (primary antibody; Sigma‒Aldrich, USA) and occludin (primary antibody; Sigma‒Aldrich, USA) were detected by Western blotting. Total protein was extracted with RIPA lysis buffer (Beyotime, Shanghai, China), and the supernatant was centrifuged to determine the protein concentration using a BCA protein quantification kit (Beyotime, Shanghai, China). The extracted proteins were denatured, isolated using 10% SDS-PAGE and moved onto polyvinylidene difluoride (PVDF) membranes. The membranes were then immersed in 5% fat-free milk mixed with TBST buffer and left to incubate for 1 h. After that, the PVDF membranes were exposed to the primary antibody overnight at 4 °C, followed by incubation with the secondary antibody at room temperature for 1 h. Subsequently, the target bands were captured using a chemiluminescent gel imager (Shanghai Tanon Technology Co., Ltd., China).

### Statistical analysis

The mean ± standard deviation (SD) is used to present data for normally distributed variables, whereas the median (interquartile range) is used for those that are not normally distributed. Groups were compared using the nonparametric Mann–Whitney U test. The false discovery rate (FDR) method was used to adjust *p* values for multiple comparisons. Associations between metabolites, microbial taxa, and kidney injury markers were assessed using Spearman’s correlation analysis; metabolites and microbes (|r| > 0.7) with statistical significance (*p* < 0.05) were subsequently analysed for further correlation analysis (Supplementary Table 1). The statistical analyses were conducted using IBM SPSS Statistics for Windows, version 23.0 (IBM Corp., Armonk, NY, USA), and R (version 4.2.1). Statistical significance was set at *p* < 0.05.

## Results

### Cisplatin-induced acute kidney injury and renal pathological changes

To evaluate the successful establishment of the cisplatin-induced acute kidney injury (AKI) model, renal function and pathological changes were assessed. Compared with those in the NC group, the body weights of the rats in the Cis group steadily increased during the 7-day adaptation period. However, after a single intraperitoneal injection of cisplatin (10 mg/kg) on Day 7, the body weights of the rats in the Cis group significantly and progressively decreased (*p* < 0.0001) until the rats were euthanized on Day 10, whereas the weights of the rats in the NC group continued to increase (Fig. 1b). This phenotype was consistent with clinical observations of cisplatin-induced anorexia and weight loss in patients with cancer. Compared with those in the normal control group (NC group), the serum creatinine (SCr) and blood urea nitrogen (BUN) levels in the cisplatin (Cis) group significantly increased (Fig. 1c). Consistent with the functional impairment, histological examination of kidney tissues via H&E staining revealed severe tubular injury in the Cis group, characterized by extensive tubular necrosis, marked epithelial cell shedding, interstitial oedema, brush border disruption and cast formation in the kidney cortex (Fig. 1d). To investigate whether the AKI induced by cisplatin was accompanied by disruption of the intestinal barrier, we detected the expression levels of the tight junction proteins ZO-1 and occludin in colonic tissue using Western blotting (Fig. 1e). Compared with those in the NC group, the protein expression levels of ZO-1 (*p* = 0.001) and occludin (*p* = 0.001) were significantly lower in the Cis group. These findings confirm that intestinal barrier disruption is a prominent feature of the systemic pathological model of cisplatin-induced AKI.

### Alterations in gut microbiota composition

**Fig. 2:**
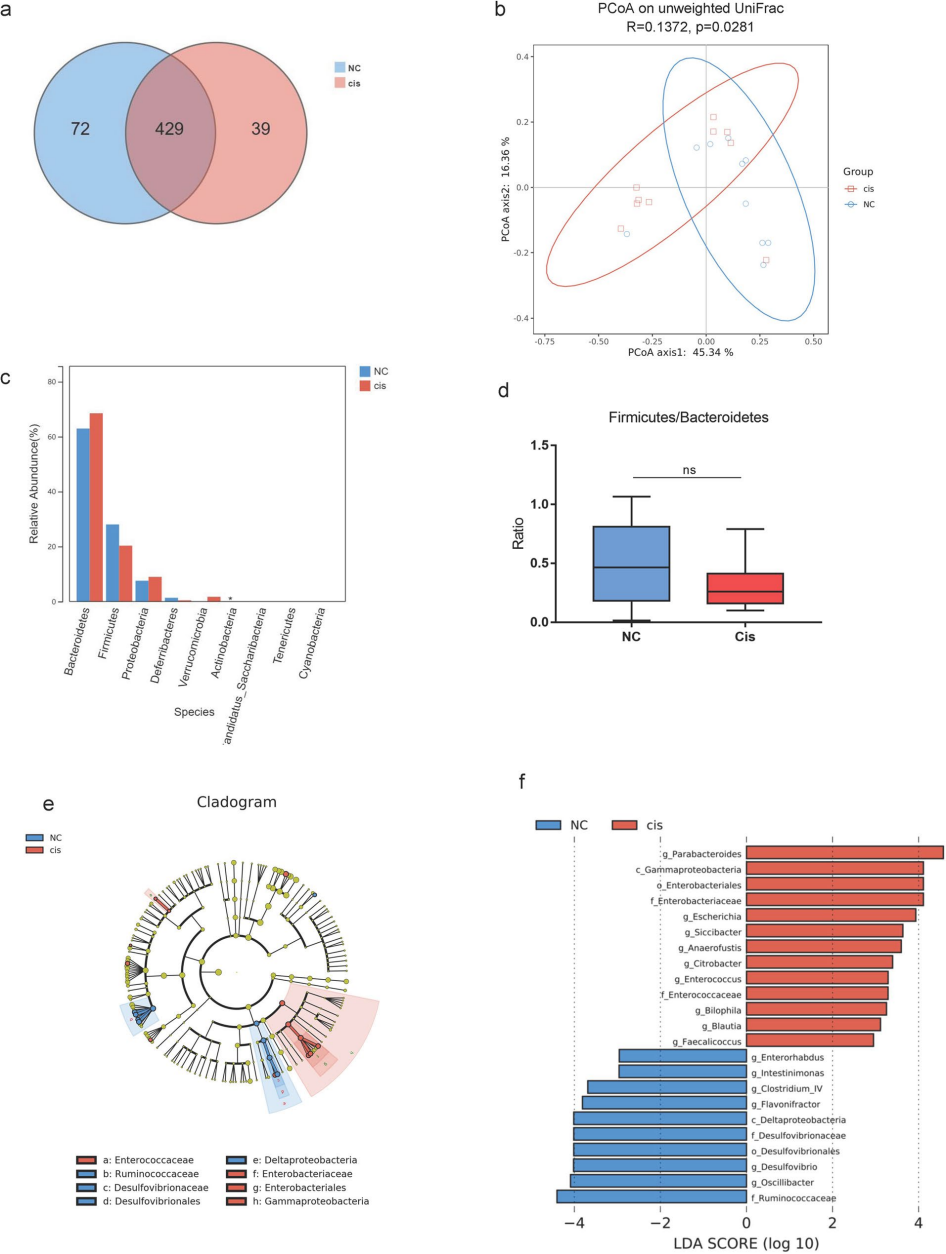
Alterations in Gut Microbiota Composition. **a** Venn diagram showing the shared and unique gut microbiota among groups. **b** Principal coordinate analysis (PCoA) plot based on unweighted UniFrac distance metrics with β-diversity analysis. **c** Bar Chart showing the comparison of microbial composition at Phylum level. **d **The ratio of relative abundances between *Firmicutes* and *Bacteroidetes. ***e** Cladogram generated by linear discriminant analysis effect size (LEfSe) analysis, indicating the specific microbial taxa enriched in the NC and Cis groups. **f** LDA (linear discriminant analysis) score histograms identified the taxa whose abundances significantly differed between the NC and Cis groups (LDA score > 2.0, *p* < 0.05). The length of the bar chart represents the magnitude of the impact of significantly different taxa. Regions in red indicate clades that were enriched in the Cis group those as opposed to in the control group, while regions in blue indicate clades that were enriched in the control group compared to those in the Cis group. PCoA, Principal coordinate analysis; LEfSe, linear discriminant analysis effect size; LDA, linear discriminant analysis. *, *p <* 0.05

A total of 20 faecal samples (*n* = 10 per group) were analysed, yielding 69,754–71,130 clean reads per sample. The rarefaction curves approached saturation, indicating sufficient sequencing depth for all the samples (Supplementary Figure S1a). A Venn diagram revealed 72 and 39 unique operational taxonomic units (OTUs) in the NC and Cis groups, respectively, with 429 OTUs shared between them (Fig. 2a). As shown by the results of the alpha diversity analysis, the Simpson, Chao1, Shannon and Sobs indices did not significantly differ between these two groups (Supplementary Figure S1b). However, principal coordinate analysis (PCoA) based on unweighted and weighted UniFrac distance metrics clearly revealed separation between the two groups, indicating a significant shift in microbial community structure (beta diversity) induced by cisplatin (Fig. 2b and Supplementary Figure S1c).

At the phylum level, the relative abundances of *Bacteroidetes* and *Firmicutes*, as well as the *Firmicutes/Bacteroidetes* ratio, were comparable between the two groups (Fig. 2c, d). Notably, the abundance of *Actinobacteria* was significantly decreased in the Cis group (*p* = 0.046; Fig. 2c).

At the genus level, significant disparities in bacterial taxa were identified (Table 1). The Cis group was enriched with 13 genera, i.e., *Allobaculum*,* Anaerofustis*,* Christensenella*,* Enterococcus*,* Anaerostipes*,* Phocaeicola*,* Parabacteroides*,* Longicatena*,* Enterocloster*,* Blautia*,* Parasutterella*,* Escherichia* and *Bilophila.* Conversely, the NC group harboured significantly higher abundances of 9 genera, i.e., *Adlercreutzia*,* Harryflintia*,* Schaedlerella*,* Lawsonibacter*,* Pseudoflavonifractor*,* Flintibacter*,* Anaerotignum*,* Vescimonas* and *Brotonthovivens.*

**Table 1:** Significantly altered gut microbiota at the genus level between the NC and Cis groups

Species on genus level	mean(Cis)	SD(Cis)	mean(NC)	SD(NC)	*p*.value (wilcox test)	FC (Cis/NC)	FDR	state
*Allobaculum*	0.06	0.09	0.00	0.00	0.035	/	0.147	Up
*Anaerofustis*	0.00	0.00	0.00	0.00	0.035	/	0.147	Up
*Christensenella*	0.00	0.00	0.00	0.00	0.035	/	0.147	Up
*Enterococcus*	0.02	0.05	0.00	0.00	0.002	19.66	0.100	Up
*Anaerostipes*	0.19	0.23	0.01	0.01	0.011	17.88	0.129	Up
*Phocaeicola*	11.04	14.43	0.95	1.43	0.031	11.60	0.147	Up
*Parabacteroides*	2.93	2.49	0.37	0.25	0.007	7.86	0.129	Up
*Longicatena*	0.08	0.10	0.01	0.02	0.020	5.73	0.147	Up
*Enterocloster*	8.37	8.87	1.49	3.41	0.014	5.63	0.129	Up
*Blautia*	0.38	0.41	0.09	0.14	0.031	3.99	0.147	Up
*Parasutterella*	1.18	0.85	0.45	0.60	0.031	2.62	0.147	Up
*Escherichia*	1.82	3.58	1.43	4.23	0.028	1.27	0.147	Up
*Bilophila*	0.56	0.47	0.48	1.24	0.014	1.19	0.129	Up
*Adlercreutzia*	0.01	0.01	0.05	0.04	0.007	0.20	0.129	Down
*Harryflintia*	0.01	0.01	0.08	0.11	0.019	0.08	0.147	Down
*Schaedlerella*	0.01	0.02	0.11	0.12	0.015	0.08	0.129	Down
*Lawsonibacter*	0.09	0.17	1.42	1.40	0.007	0.07	0.129	Down
*Pseudoflavonifractor*	0.00	0.00	0.01	0.03	0.028	0.06	0.147	Down
*Flintibacter*	0.16	0.25	2.92	2.40	0.011	0.05	0.129	Down
*Anaerotignum*	0.01	0.01	0.13	0.15	0.045	0.04	0.180	Down
*Vescimonas*	0.03	0.07	0.87	1.50	0.035	0.04	0.147	Down
*Brotonthovivens*	0.02	0.06	0.62	1.15	0.002	0.03	0.100	Down

Up indicates higher abundance in the Cis group; Down indicates lower abundance in the Cis group

Linear discriminant analysis effect size (LEfSe) further revealed specific bacterial taxa enriched in each group (LDA score > 2.0, *p <* 0.05). The Cis group was enriched with *g_Parabacteroides*,* c_Gammaproteobacteria*,* o_Enterobacteriales*,* f_Enterobacteriaceae*,* g_Escherichia*,* g_Siccibacter*,* g_Anaerofustis*,* g_Citrobacter*,* g_Enterococcus*,* f_Enterococcaceae*,* g_Bilophila*,* g_Blautia*, and *g_Faecalicoccus.* In contrast, the NC group was predominantly enriched in *g_Enterorhabdus*,* g_Intertinimonas*,* g_Clostridium_IV*,* g_Flavonifractor*,* c_Deltaproteobacteria*,* f_Desulfovibrionaceae*,* o_Desulfovibrionales*,* g_Desulfovibrio*,* g_Oscillibacter*, and *f_Ruminococcaceae* (Fig. 2e, f).

To explore the differences in microbial functions between the NC and Cis groups, we performed functional prediction using PICRUSt based on the KEGG database-suggested alterations in microbial metabolic pathways in the Cis group (Supplementary Figure S2).

### Faecal metabolic profile analysis

**Fig. 3:**
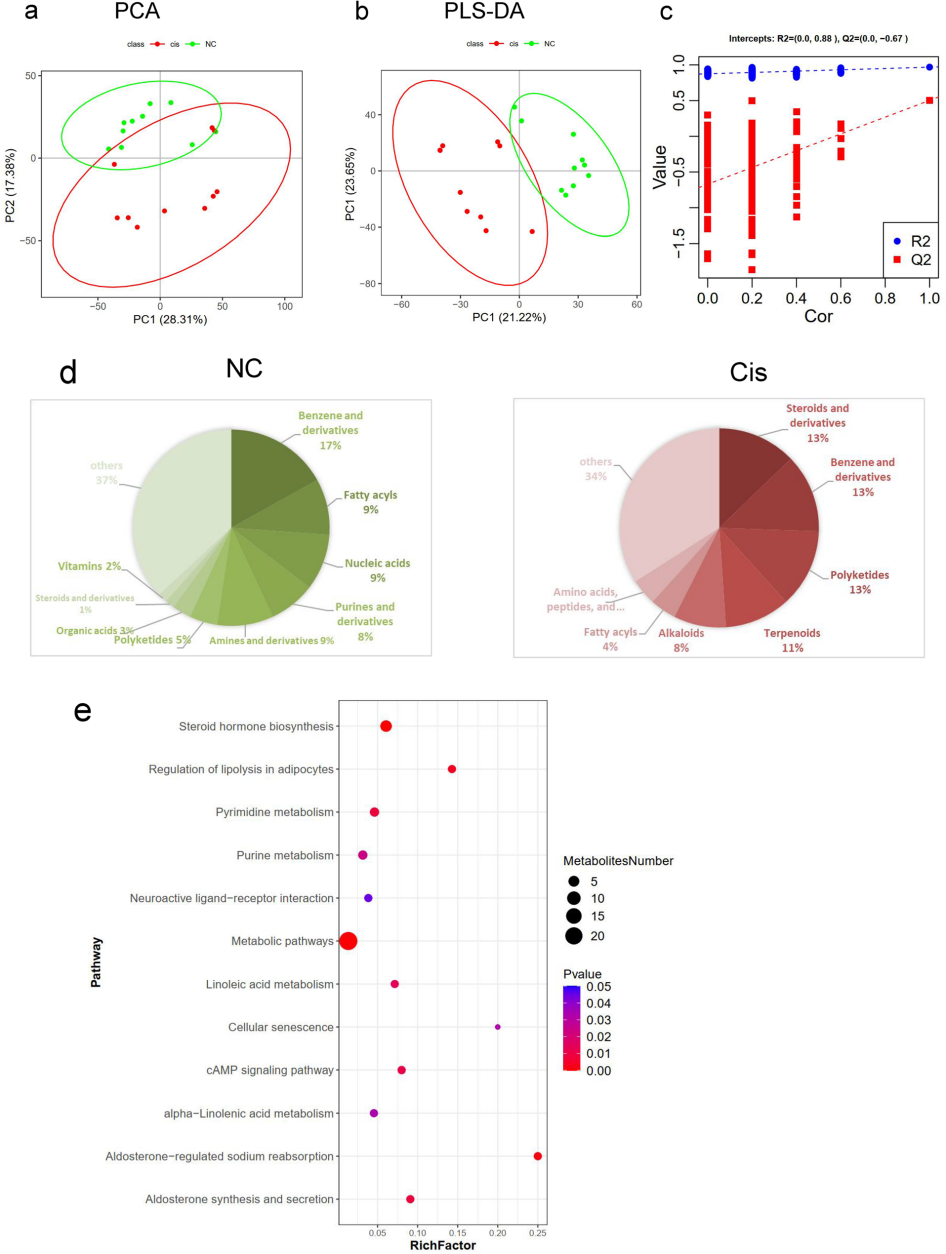
Fecal Metabolic Profile Analysis. **a** Principal component analysis (PCA) score plot for the fecal metabolites between the NC and Cis groups. **b** Partial least squares discriminant analysis (PLS-DA) score plot for the fecal metabolites between the NC and Cis groups. **c** PLS-DA permutation test evaluating the robustness and significance of the discriminant model. **d** Fecal metabolite class distribution in NC and Cis groups. **e** Enrichment analysis of differential metabolites in the KEGG pathway. PCA, Principal Component Analysis; PLS-DA, Partial Least Squares Discriminant Analysis; KEGG, Kyoto Encyclopedia of Genes and Genomes

Untargeted LC‒MS/MS-based metabolomic analysis revealed a distinct separation between the Cis and NC groups according to their PCA score plots, demonstrating a profound perturbation of the faecal metabolome in AKI rats (Fig. 3a). This distinction was further confirmed by partial least squares-discriminant analysis (PLS-DA) (Fig. 3b). The intercept-related R² values consistently approximated 0.88 across all levels of correlation (Cor), suggesting a robust model fit. The Q² values exhibited a monotonic increase from − 1.5 to − 0.67 as the Cor values rose from 0 to 1.0, indicating a gradual increase in the model’s predictive performance with increasing correlation among the independent variables (Fig. 3c). The robustness of the PLS-DA model was validated through 7-fold cross-validation and permutation testing. Based on a threshold of variable importance in projection (VIP) > 1.5 and *p <* 0.05, 20 named differentially abundant metabolites were detected (Table 2).

**Table 2:** Significantly altered metabolites between the NC group and the Cis group

Metabolite	VIP	FC	FDR	State
Styrene	2.48	73.32	0.025	Up
Sirolimus	2.37	45.02	0.024	Up
Dehydroepiandrosterone (dhea)	2.32	19.46	0.019	Up
D-urobilin	1.97	16.23	0.022	Up
Tetrahydrocortisone	2.19	14.12	0.024	Up
Corticosterone	2.06	8.21	0.029	Up
Cortisone	2.12	8.16	0.044	Up
5,6-dihydroxy-2-indolecarboxylic acid	1.84	5.31	0.019	Up
Formylkynurenine	1.56	4.61	0.028	Up
Aldosterone	1.82	3.73	0.036	Up
N-acetylaspartic acid	1.81	3.09	0.031	Up
Linoleic acid	1.61	0.39	0.007	Down
Adenosine	1.97	0.26	0.025	Down
Histamine	2.22	0.23	0.011	Down
12-oxo phytodienoic acid	1.97	0.22	0.005	Down
Vitamin d2	2.15	0.20	0.024	Down
Guanine	2.14	0.17	0.007	Down
Indole-3-acetaldehyde	1.76	0.14	0.042	Down
Tocopheryl acetate	2.54	0.13	0.012	Down
4-toluic acid	1.81	0.09	0.039	Down

Metabolites were selected based on a Variable Importance in Projection (VIP) score > 1.5 (from PLS-DA) and a false discovery rate (FDR)-adjusted *p*-value < 0.05. (Up indicates higher abundance in the Cis group; Down indicates lower abundance in the Cis group)

Pie charts were constructed to depict the compositional distribution of the faecal metabolite classes in the NC and Cis groups. In the NC group, the most prevalent metabolite classes included benzene and derivatives (17%), amines and derivatives (9%), fatty acyls (9%), and nucleic acids (9%), whereas steroids and derivatives were relatively uncommon (1%). In contrast, the Cis group exhibited a shift in the metabolite profile, characterized by increased representations of steroids and derivatives (13%), benzene and derivatives (13%), polyketides (13%), and terpenoids (11%). Alkaloids (8%) and fatty acyls (4%) were also prominent, whereas the proportions of amines and derivatives, as well as purines and derivatives, decreased. These alterations suggest significant cisplatin-induced modification of the faecal metabolome, particularly marked by increases in the levels of steroids, polyketides, and terpenoid metabolites and decreases in the levels of amine and purine derivatives (Fig. 3d).

KEGG pathway enrichment analysis revealed 12 significantly altered pathways (*p <* 0.05) (Fig. 3e). Among these pathways, steroid hormone biosynthesis, metabolic pathways, aldosterone-regulated sodium reabsorption, regulation of lipolysis in adipocytes, pyrimidine metabolism and aldosterone synthesis and secretion were the six pathways with the most significant enrichment (*p <* 0.01, Supplementary Figure S2). We focused on 20 metabolites with VIP > 1.5, including guanine (fold change, FC = 0.17), histamine (FC = 0.23), tocopheryl acetate (FC = 0.13), dehydroepiandrosterone (DHEA; FC = 19.46), vitamin d2 (FC = 0.20), tetrahydrocortisone (FC = 14.12), sirolimus (FC = 45.02), styrene (FC = 73.32), corticosterone (FC = 8.21), and cortisone (FC = 8.16). These 20 faecal metabolites were identified as characteristic metabolites for Cis rats.

### Correlations among the gut microbiota, metabolites, and renal function

**Fig. 4:**
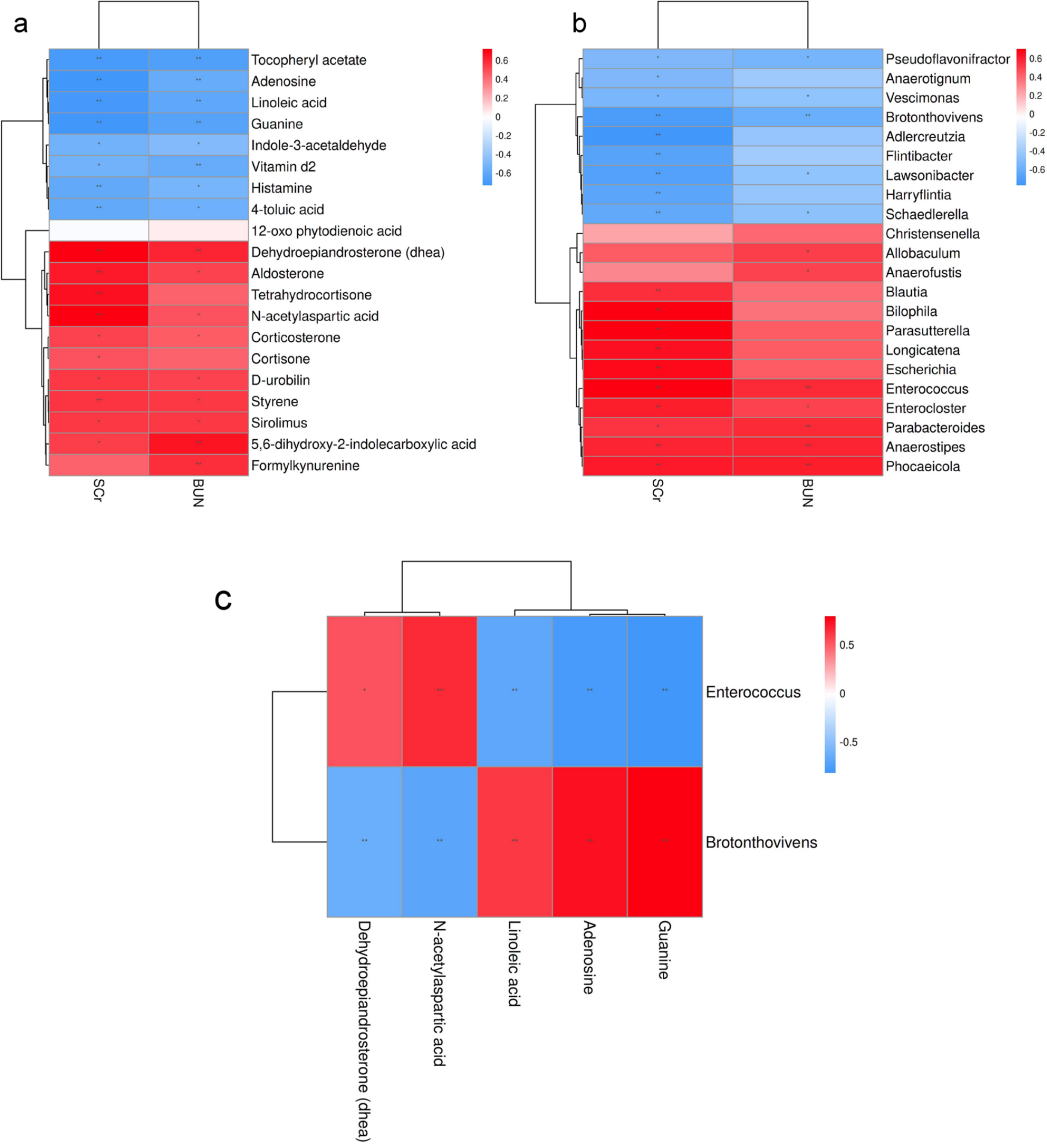
Correlations among Gut Microbiota, Metabolites, and Renal Function. **a **Heatmap showing the correlations between fecal metabolites and important indicators of renal insufficiency (SCr and BUN). **b** Heatmap showing the correlations between the gut microbiota and important indicators of renal insufficiency. **c** Heatmap showing the correlations between differential fecal metabolites and gut microbiota. * indicates microbiota significantly correlated with metabolites (Spearman’s correlation analysis, *p <* 0.05). Red indicates positive correlations, while blue indicates negative correlations. SCr, serum creatinine; BUN, blood urea nitrogen

Spearman correlation analysis (|r| > 0.7) was then performed to investigate the relationships between differentially abundant metabolites and renal function markers (SCr and BUN). Three faecal metabolites, adenosine (*r* = − 0.716), guanine (*r* = − 0.728) and linoleic acid (*r* = − 0.710), were negatively correlated with SCr and BUN levels, whereas DHEA (*r* = 0.722) and N-acetylaspartic acid (NAA; *r* = 0.713) were positively correlated with these renal injury markers (Fig. 4a).

Analysis of the gut microbiota–renal function axis using the same analytical approach revealed a significant positive correlation between the genus *Enterococcus* and the serum creatinine (SCB)/BUN ratio (*r* = 0.701). Conversely, *Brotonthovivens* (*r*=-0.723) was negatively correlated with the SCr/BUN ratio (Fig. 4b).

To further elucidate the interplay between the gut microbiota and faecal metabolites, a correlation heatmap was constructed based on the results of Spearman’s correlation analysis (Fig. 4c). *Enterococcus* was positively correlated with DHEA and NAA but was significantly negatively correlated with linoleic acid, adenosine, and guanine. In contrast, *Brotonthovivens* was strongly positively correlated with linoleic acid, adenosine, and guanine and negatively correlated with DHEA and NAA.

### Metabolite modulation of cisplatin-induced cytotoxicity and apoptosis in HK-2 cells

To investigate the correlation between specific metabolites and cisplatin-induced AKI, we established an in vitro model of cisplatin injury in human renal proximal tubular epithelial (HK-2) cells and evaluated the mechanistic roles of adenosine, linoleic acid, and NAA. These three metabolites were selected because of their documented associations with kidney disease in previous studies involving animal and cell models. In contrast, DHEA, a hormone with potential side effects, and guanine, which lacks relevant literature on renal disease, were excluded from our study.

**Fig. 5:**
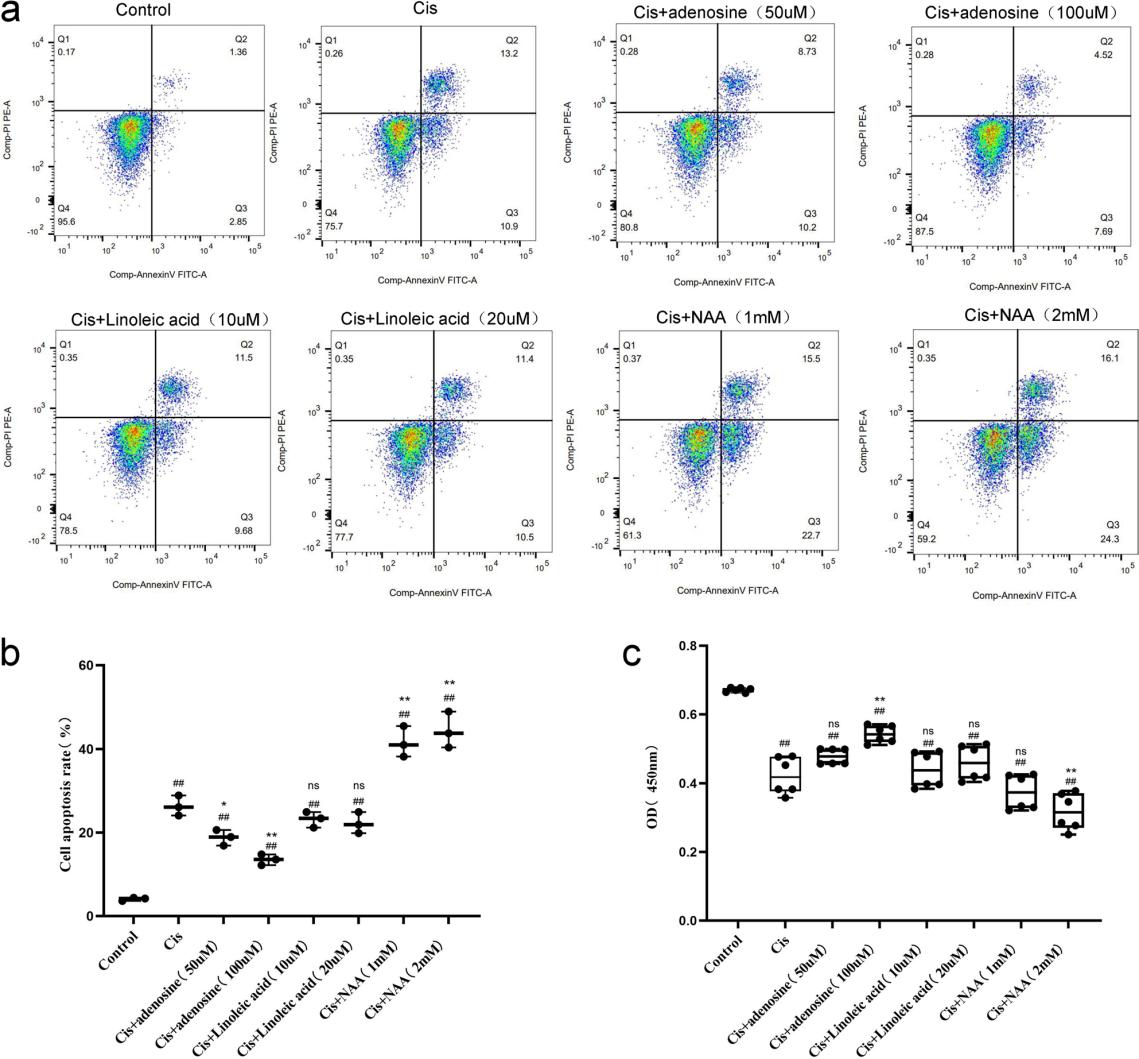
Metabolite Modulation of Cisplatin-Induced Cytotoxicity and Apoptosis in HK-2 Cells. **a**, **b** HK-2 cell apoptosis rates after treatment with cisplatin alone or combined with adenosine, linoleic acid, or NAA, assessed by flow cytometry (*n* = 3). **c** HK-2 cell viability under the same treatments, measured by CCK-8 assay (*n* = 6). Data are mean ± SD. #*p* < 0.05, ##*p* < 0.01 vs. Control; *p < 0.05, **p < 0.01 vs. Cisplatin group; *ns* = not significant (*p* ≥ 0.05)

Flow cytometric analysis confirmed that compared with the control treatment, cisplatin treatment significantly increased apoptosis in HK-2 cells (*p* < 0.01). Treatment with adenosine suppressed apoptosis in a concentration-dependent manner, with 50 µM and 100 µM adenosine reducing apoptosis rates (*p* < 0.05 and *p* < 0.01 vs. the cisplatin group, respectively). Conversely, NAA cotreatment (1 mM and 2 mM) markedly increased apoptosis compared with that in the cisplatin group (*p* < 0.01 for both concentrations) (Fig. 5a, b).

CCK-8 assays revealed that compared with the control treatment, treatment with cisplatin (20 µM) significantly reduced HK-2 cell viability (0.42 ± 0.02 vs. 0.67 ± 0.006; *p* < 0.01). Treatment with adenosine attenuated this cytotoxicity in a concentration-dependent manner, with 100 µM adenosine demonstrating a statistically significant protective effect (0.54 ± 0.025 vs. the cisplatin group; *p* < 0.01). In contrast, NAA cotreatment exacerbated cisplatin-induced injury: 2 mM NAA further reduced cell viability (0.32 ± 0.05 vs. the cisplatin group; *p* < 0.01), indicating that NAA potentiates cisplatin-mediated cytotoxicity (Fig. 5c).

In summary, in an in vitro HK-2 cell model, adenosine mitigated cisplatin-induced cytotoxicity and suppressed apoptosis in a concentration-dependent manner, thereby exerting a renoprotective effect. Conversely, NAA markedly exacerbated cisplatin-mediated injury and apoptosis in renal tubular epithelial cells, indicating its role as a potential pro-injury metabolite in cisplatin-induced AKI. These findings illuminate the distinct regulatory functions of key metabolites in cisplatin-induced renal tubular epithelial cell injury, offering experimental evidence to support future targeted metabolite interventions in cisplatin-induced AKI.

## Discussion

**Fig. 6:**
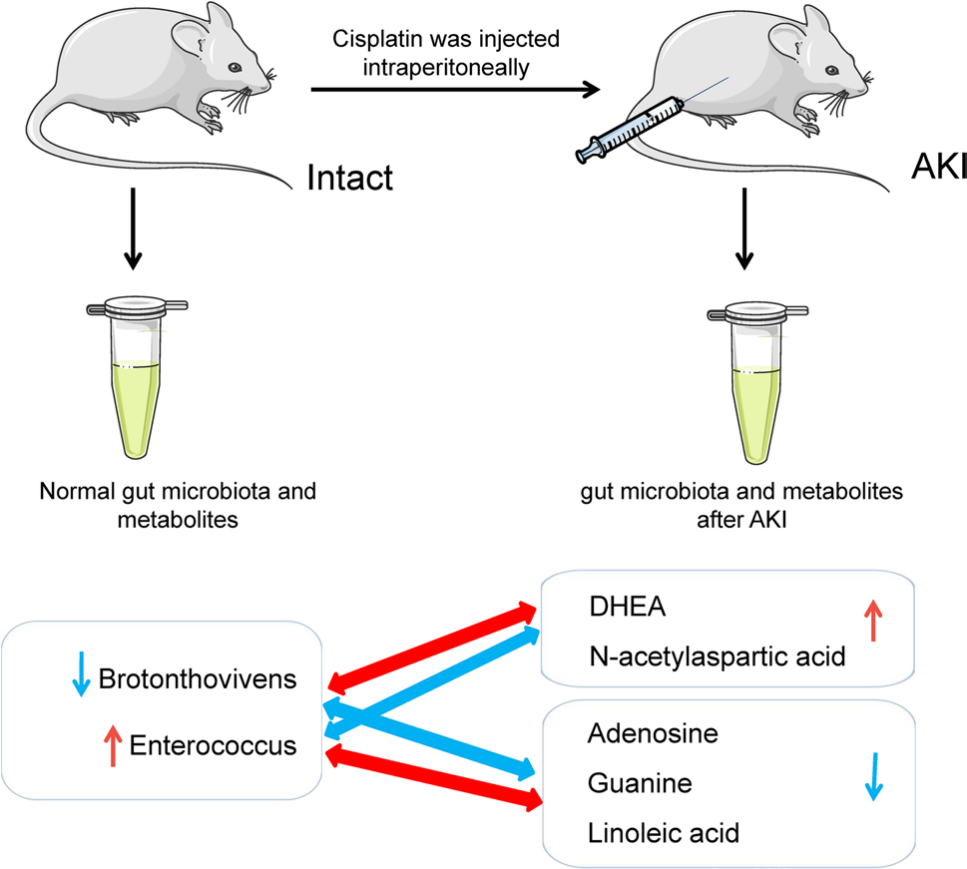
Graph Abstract. Cisplatin was administered intraperitoneally to induce AKI. Fecal samples were collected to assess the gut microbiome and metabolome. The correlations between the relative abundances of specific bacterial taxa and metabolite levels are shown. The bidirectional arrows between microorganisms and metabolites indicate a positive correlation (red) or negative correlation (blue) between them. The unidirectional arrows (all pointing to AKI) represent a positive correlation (red, upward) or negative correlation (blue, downward) between microorganisms/metabolites and the severity of AKI

By integrating 16 S rRNA sequencing and untargeted metabolomics, this study delineates the complex interplay between gut microbiota dysbiosis, metabolic disturbances, and renal dysfunction in a rat model of cisplatin-induced AKI. Our findings demonstrate that cisplatin nephrotoxicity extends beyond direct tubular injury to encompass a profound disruption of gut microbial homeostasis and associated metabolic pathways, which may collectively exacerbate renal damage. A schematic summary of these key findings is presented in Fig. 6.

Cisplatin-induced systemic toxicity, exemplified by weight loss or a lack of appetite, is a probable contributor to the observed dysbiosis and metabolite changes, which are consistent with clinical observations, such as weight loss or physical changes such as fatigue, vomiting, nausea, and a lack of appetite [21, 22]. Although it is difficult to separate the effects of direct cytotoxicity, AKI, and systemic metabolic changes in this model, the strong correlations we identified between specific bacteria (*Enterococcus*,* Brotonthovivens*), metabolites (DHEA, linoleic acid), and renal function markers remain highly meaningful. Even if dysbiosis initially arises from nonspecific stress, these pathways may play roles in the progression of renal injury.

Direct comparisons with human studies are currently limited by a lack of data specifically concerning the effects of cisplatin on the microbiome in patients [23]. The critical confounding factors that may influence cisplatin-induced microbial dysbiosis in patients are (1) polypharmacy, including antibiotics, proton pump inhibitors (PPIs), and opioids, which independently affect the microbiome [24]; (2) diet and nutrition, characterized by variable intake and cachexia, which alter the intestinal microenvironment [25]; (3) tumour-related factors, such as type, location, and surgical interventions [20]; and (4) host heterogeneity, encompassing genetic variations and baseline microbiome composition. Our controlled rodent model demonstrated a core dysbiotic shift induced by cisplatin, which in patients is further complicated and modified by these multifactorial variables.

Cisplatin treatment induced significant shifts in the gut microbiota composition, which is consistent with the established literature on kidney injury reshaping the gut microbial community [26]. At the genus level, although the FDR-adjusted *p*-values for most bacterial genera were greater than 0.05, LEfSe analysis identified several dominant taxa with LDA score > 2.0, including *Enterococcus*, indicating their significant discriminatory abundance patterns in cisplatin-induced AKI. Specifically, cisplatin administration was associated with enrichment of proinflammatory genera, such as *Enterococcus* and *Anaerofustis*, and depletion of potentially beneficial taxa, particularly *Brotonthovivens*. Structural divergence suggests that cisplatin exposure selectively modulates microbial communities, potentially disrupting intestinal homeostasis and contributing to systemic inflammation and renal injury, although a causal relationship has yet to be proven.

Notably, we identified specific associations between gut microbial genera and clinical indicators of renal injury. Only a limited subset of genera showed significant correlations with renal function markers. SCr and BUN were positively correlated with conditionally pathogenic bacteria, such as *Enterococcus*, which is consistent with previous reports in AKI models [27, 28]. *Enterococcus* enrichment is frequently associated with proinflammatory and pathogenic activities, including disruption of the epithelial barrier and the induction of cytokines, which may exacerbate renal inflammation [29]. Although *Anaerostipes* did not meet the threshold for a strong correlation with SCr or BUN in this study, it has been reported to be reduced in patients with chronic kidney disease [30]. The discrepant patterns observed between acute and chronic kidney injury may reflect stage-specific host–microbiota interactions or differences in inflammatory and metabolic states rather than a direct contribution of *Anaerostipes* to acute renal injury. In contrast, a higher abundance of *Brotonthovivens* was correlated with lower SCr and BUN levels, suggesting a potential protective role. Nevertheless, given the scarcity of studies on this genus, the inverse association with renal injury markers that was observed here highlights the need for further investigation. These findings underscore the potential of microbes as biomarkers for cisplatin-induced nephrotoxicity.

Metabolomic profiling further revealed that specific faecal metabolites were intricately associated with renal function, reflecting alterations in metabolic, hormonal, and filtration pathways [31–34]. Among the differentially abundant metabolites, adenosine, guanine, and linoleic acid were negatively correlated with SCr/BUN. Our subsequent in vitro validation using HK-2 cells provided direct functional evidence supporting these metabolomic associations. Specifically, adenosine supplementation attenuated cisplatin-induced cytotoxicity and apoptosis in a concentration-dependent manner, confirming its direct renoprotective role beyond mere correlation. These findings align with the established roles of adenosine in cellular energy salvage and redox homeostasis [35, 36]. During AKI, the downregulation of adenosine may impair adaptive stress response mechanisms in renal tubular cells [37, 38]. Similarly, the inverse association of linoleic acid with renal dysfunction is consistent with its anti-inflammatory properties, although direct experimental evidence in our HK-2 model remains lacking. Future studies should test whether linoleic acid cotreatment recapitulates the protective effects of adenosine, particularly given its role in mitigating oxidative tubular injury [39].

In contrast, and in strong support of the results of the metabolomic prediction, our cell experiments demonstrated that NAA significantly exacerbated cisplatin-induced HK-2 cell damage and apoptosis. This finding directly translates the observed positive correlation between NAA levels and renal dysfunction into a causal, deleterious effect at the cellular level. Cisplatin-induced oxidative stress is likely correlated with elevated NAA levels, reflecting impaired mitochondrial energy production and amino acid catabolism [40, 41]. Notably, DHEA exhibits a paradoxical association with worsened renal injury despite its established renoprotective role in models of ischaemic/diabetic kidney injury [42–44], underscoring the context-dependent nature of metabolite functions. We propose that cisplatin-induced oxidative stress subverts the protective effect of DHEA by disrupting adrenal synthesis and converting it into a maladaptive stress signal. Together, these metabolic alterations may represent a compensatory but ultimately insufficient response to tubular injury, contributing to renal dysfunction.

Integrative analysis of microbiome and metabolome data elucidated intricate associations between specific bacterial taxa and altered metabolites [45]. Notably, diametrically opposite correlation patterns were observed for *Enterococcus* and *Brotonthovivens*, with key metabolites linked to renal function. *Enterococcus* was positively correlated with DHEA and NAA but negatively correlated with linoleic acid, adenosine, and guanine. Conversely, *Brotonthovivens* displayed an inverse correlation pattern. This stark contrast suggests that these genera occupy complementary niches and exert opposing influences on host metabolic homeostasis and renal pathophysiology.

The observed negative correlation between *Enterococcus* and beneficial metabolites such as linoleic acid, adenosine, and guanine may reflect active microbial consumption or contextual dysregulation. Host-derived unsaturated fatty acids, such as linoleic and oleic acids, can be incorporated into bacterial membranes and enhance the resilience of *Enterococcus faecalis* against environmental stress [46, 47]. Furthermore, under stress or unfavourable environmental conditions, *Enterococcus* can remodel its amino acid and nucleotide metabolism—particularly involving adenosine and guanine—to sustain growth and facilitate biofilm formation [48, 49]. However, direct evidence linking *Enterococcus* to DHEA or NAA remains intriguing. *Enterococcus* and its metabolites remain limited and warrant further investigation.

Conversely, the correlation pattern of *Brotonthovivens* aligns with a potentially renoprotective phenotype. Its positive association with metabolites involved in purine salvage (adenosine, guanine) and fatty acid metabolism (linoleic acid) implies a role in the maintenance of energy balance and redox stability—processes crucial for renal cellular integrity [50, 51]. These findings provide further support for the concept that specific microbes, through their capacity to produce, modify, or consume key host metabolites, may influence renal outcomes via the gut–kidney axis.

This study elucidates the potential bidirectional crosstalk within the gut–kidney axis during cisplatin-induced AKI. Western blot analysis demonstrated that cisplatin significantly downregulated the expression levels of the colonic tight junction proteins ZO-1 and occludin in rats, suggesting that the intestinal barrier was impaired and that the permeability was increased, which is correlated with the pathogenesis of AKI. Although our correlative data do not establish renal injury as the primary instigator of intestinal dysfunction, complementary experiments with HK-2 cells confirmed the direct regulatory effects of key differentially abundant metabolites on renal tubular cell injury and apoptosis, thereby providing experimental evidence for metabolite-targeted interventions. This research underscores the critical clinical objective of mitigating cisplatin-induced nephrotoxicity—a primary cause of early treatment discontinuation—while maintaining its well-documented antitumour efficacy across various malignancies. Future time-course studies examining intestinal permeability, microbial translocation, and metabolite dynamics are essential for elucidating the causal mechanisms underlying this potential reversal of the gut–kidney axis, laying a robust foundation for the translation of these findings into clinical strategies.

Despite the inherent complexities, our findings offer a foundation for developing testable interventions targeting the gut–kidney axis to alleviate cisplatin toxicity, which are proposed for future clinical pilot studies. These interventions include the following: (1) targeted biotic therapies, such as the administration of probiotics (e.g., Lactobacillus and Bifidobacterium strains), to counteract Enterococcus or enhance barrier function [28, 52]; (2) dietary modifications, specifically the enrichment of polyunsaturated fatty acids (e.g., linoleic acid), which were negatively correlated with renal injury in our model during cisplatin therapy [53]; and (3) gut barrier support through the adjunctive use of barrier-protective agents (e.g., glutamine and zinc) to counteract the downregulation of ZO-1 and occludin expression, thereby reducing microbial translocation [54, 55].

Several limitations of this study should be noted. First, the relatively small sample size (10 rats per group) may have constrained the statistical power and broader applicability of the results. Second, this preclinical rat model of acute drug-induced injury cannot fully replicate the complex pathological context of human AKI (e.g., comorbidities, polypharmacy, genetic heterogeneity), which may restrict direct clinical translation. Third, although Western blot analysis of ZO-1 and occludin confirmed significantly impaired intestinal barrier integrity in the Cis group, we did not thoroughly evaluate systemic inflammation, microbial translocation dynamics, or their direct causal associations with renal injury. These are key mediators that may collectively modulate the observed microbial and metabolic alterations and require further elucidation. Fourth, there is an inherent distinction between faecal and blood metabolite profiles and quantification. Faecal metabolites were chosen because of their ability to directly reflect intestinal metabolic shifts and dietary influences, thereby supporting translatable dietary and prebiotic interventions. In contrast, blood metabolites provide a more accurate representation of systemic metabolic and mechanistic changes. The absence of paired blood data constrains a comprehensive understanding of cisplatin-induced metabolic dysregulation between the gut and kidneys. Furthermore, this study utilized only 16 S rRNA sequencing and did not incorporate metagenomics, which limits the ability to conduct an in-depth exploration of microbial functional changes and microbe‒metabolite interactions. Notably, our findings are correlative; owing to the broadly nonsignificant microbial differences observed, our focus was on metabolite‒renal cell interactions. Future research employing germ-free models, targeted interventions, and metagenomic analyses is necessary to establish definitive causality.

## Conclusion

In conclusion, our multiomics analysis revealed a critical interplay between specific gut microbiota members and associated metabolic disturbances in the pathogenesis of cisplatin-induced AKI. These findings extend our understanding of cisplatin nephrotoxicity beyond direct tubular injury, revealing that the gut–kidney axis is a key contributor to disease severity, likely through the modulation of systemic metabolism. The results of the present study provide insights into cisplatin-induced dysregulation of the gut flora and metabolites in AKI and may contribute to the understanding of gut flora-mediated pathophysiological mechanisms. 

## Supplementary Information

Supplementary Material 1: Supplementary Figure 1. (a) Shannon-Wiener curves of 20 fecal samples. (b) The comparison of gut microbiota α-diversity between each group, including the Simpson, Chao1, Shannon and Sobs indices.

Supplementary Material 2: Supplementary Figure 2. Prediction of functional potentials and pathway differences based on 16S rRNA sequencing data using PICRUSt with reference to the KEGG database. (a) Bar plot showing the differential abundance of KEGG pathways at Level 1 between the NC and Cis groups. (b) Significantly altered KEGG pathways at Level 2.(c) Detailed list of significantly enriched or depleted KEGG pathways at Level 3, ranked by statistical significance. KEGG, Kyoto Encyclopedia of Genes and Genomes; PICRUSt, Phylogenetic Investigation of Communities by Reconstruction of Unobserved States.

Supplementary Material 3: Supplementary Table 1. Spearman correlation coefficients (r) and p-values for the associations depicted in Figure 4.This Excel file contains six sheets providing the full statistical results (r and p-values) for the correlation analyses shown in Figure 4, including relationships between gut microbiota, fecal metabolites, and renal function markers (SCr and BUN). SCr, serum creatinine; BUN, blood urea nitrogen.

## Data Availability

The raw 16 S rRNA amplicon sequencing data generated in this study have been deposited in the NCBI Sequence Read Archive (SRA) under BioProject accession numbers PRJNA1376479.

## Abbreviations

AKI: Acute kidney injury
UPLC-MS/MS: Untargeted metabolomics
SCr: Serum creatinine
BUN: Blood urea nitrogen
CCK-8: Cell Counting Kit-8
DHEA: Dehydroepiandrosterone
NAA: N-acetylaspartic acid
SCFAs: Short-chain fatty acids
RT: Retention time
PQN: Probabilistic Quotient Normalization
PCA: Principal component analysis
PLS-DA: Partial least squares discriminant analysis
VIP: Variable important in projection
OTUs: Unique operational taxonomic units
PCoA: Principal coordinates analysis
LEfSe: Linear discriminant analysis effect size
KEGG: Kyoto Encyclopedia of Genes and Genomes
PICRUSt: Phylogenetic Investigation of Communities by Reconstruction of Unobserved States
