# Supplementary figures and images for: Gut microbiota–metabolite interactions in cisplatin-induced acute kidney injury in rats
Source: BMC Microbiol. 2026 Mar 11;26:377. doi: 10.1186/s12866-026-04927-7 (PMC13088379; doi:10.1186/s12866-026-04927-7)

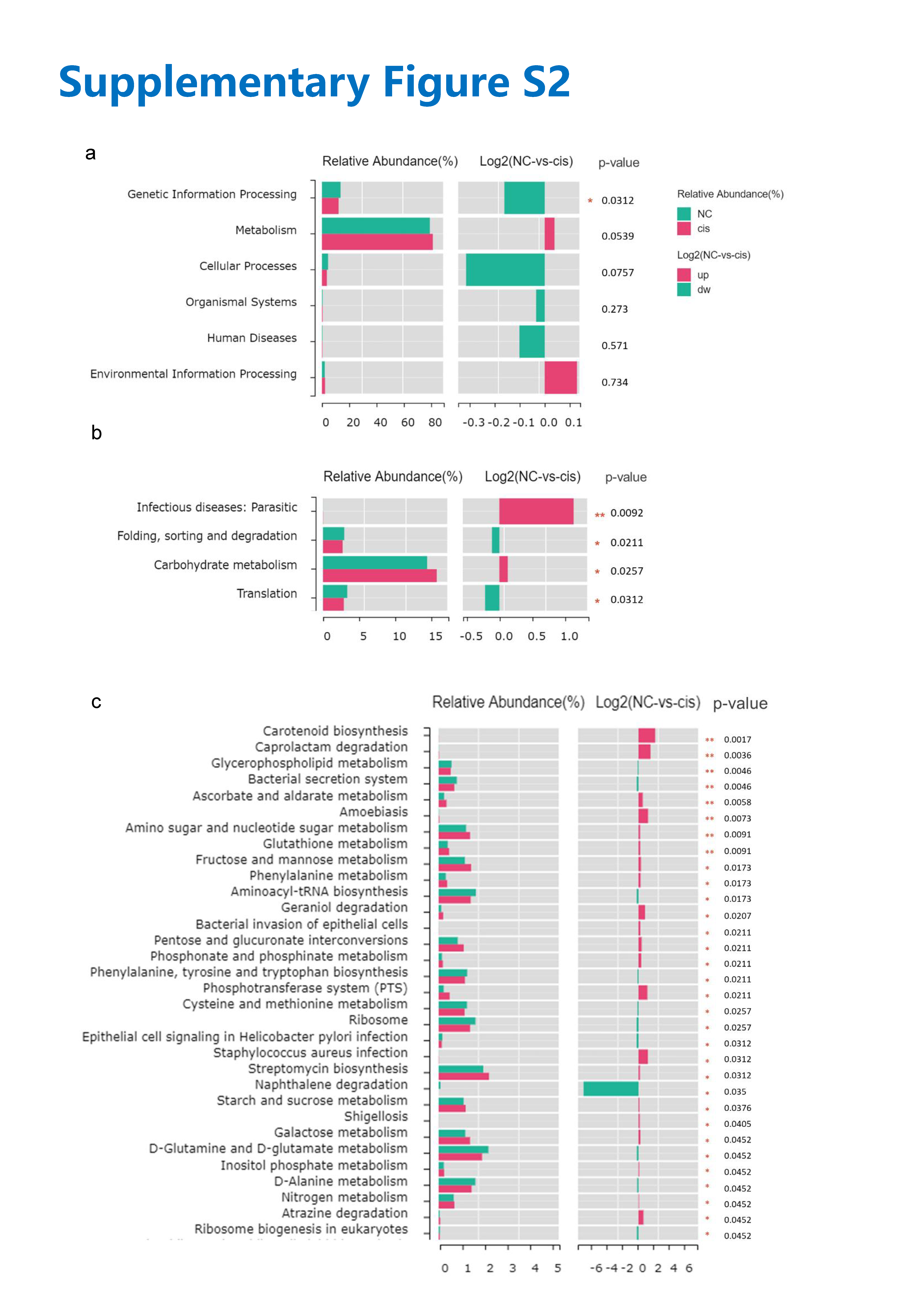

Supplement: Supplementary file 1 — Supplementary Material 1: Supplementary Figure 1. (a) Shannon-Wiener curves of 20 fecal samples. (b) The comparison of gut microbiota α-diversity between each group, including the Simpson, Chao1, Shannon and Sobs indices. [file 12866_2026_4927_MOESM1_ESM.tif]

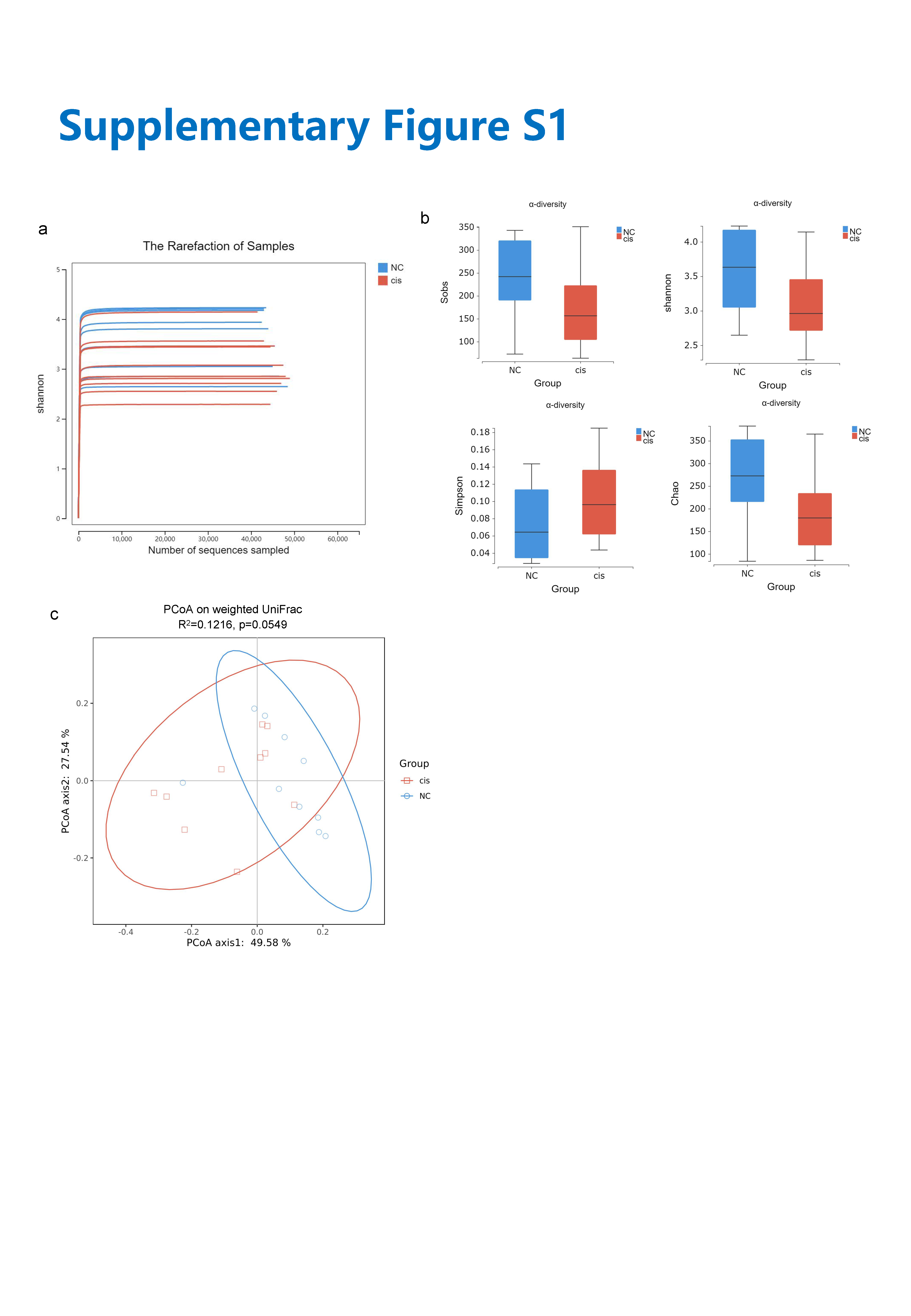

Supplement: Supplementary file 3 — Supplementary Material 3: Supplementary Table 1. Spearman correlation coefficients (r) and p-values for the associations depicted in Figure 4.This Excel file contains six sheets providing the full statistical results (r and p-values) for the correlation analyses shown in Figure 4, including relationships between gut microbiota, fecal metabolites, and renal function markers (SCr and BUN). SCr, serum creatinine; BUN, blood urea nitrogen. [file 12866_2026_4927_MOESM3_ESM.tif]
